# Three-Year Follow-Up of Posterior Corneal Elevation in Thin Corneas After Small Incision Lenticule Extraction

**DOI:** 10.3389/fmed.2022.758223

**Published:** 2022-02-04

**Authors:** Yu Zhao, Dan Fu, Zhuoyi Chen, Xingtao Zhou

**Affiliations:** ^1^Department of Ophthalmology and Optometry, Eye and ENT Hospital of Fudan University, Shanghai, China; ^2^NHC Key Laboratory of Myopia (Fudan University), Shanghai, China; ^3^Laboratory of Myopia, Chinese Academy of Medical Sciences, Shanghai, China; ^4^Shanghai Research Center of Ophthalmology and Optometry, Shanghai, China

**Keywords:** posterior corneal elevation, thin cornea, SMILE, Pentacam, corneal stability

## Abstract

**Purpose:**

To evaluate the changes in posterior corneal elevation in thin corneas after small incision lenticule extraction (SMILE).

**Methods:**

In this prospective study, 97 eyes of 97 patients undergoing SMILE were recruited. Eyes were categorized into the following groups based on the preoperative minimum central corneal thickness (CCT): group A (37 eyes, 480–499 μm), group B (30 eyes, 500–529 μm), and group C (30 eyes, 530–560 μm). The posterior corneal surface was measured with a Pentacam over a 3-year follow-up period. Changes in the posterior corneal elevation at the central point (PCE), thinnest point (PTE), and predetermined area were measured.

**Results:**

No iatrogenic keratectasia was observed during the follow-up period. The mean changes in PCE, PTE, and the inferior area in group A were 1.14 ± 3.40 μm, −0.11 ± 3.20 μm, and −0.26 ± 1.23 μm, respectively (*P* ≥ 0.125). Although statistically significant change in the central-4 mm area was noted, the value was quite small (0.98 ± 1.67 μm) and was not higher than that in the other two groups (*P* = 0.003). For all three groups, the elevation remained stable or showed a backward change in the central annulus, while there was a small forward displacement in the 6-mm optical zone. In group A, changes in elevation values yielded negative statistical correlations with residual bed thickness and CCT (*P* ≤ 0.006) (except for the inferior area, the 4-mm and 6-mm optical zone).

**Conclusions:**

With a strict preoperative assessment, SMILE achieved good safety and efficacy in correcting myopia in thin corneas and enabled a stable posterior corneal surface over a 3-year follow-up period.

**Synopsis:**

Careful preoperative assessment and suitable surgical design should be taken to ensure posterior corneal stability after SMILE in thin corneas.

## Introduction

Since Professor Seiler first reported three cases of iatrogenic keratectasia after laser in situ keratomileusis (LASIK) in 1998, this severe postoperative complication has gained worldwide attention both from ophthalmologists and patients (1). Although the prevalence is relatively low, it could cause severely decreased visual acuity, perpetual corneal thinning, and protrusion; in some extreme cases, the patients may need a corneal transplant (2, 3). Theoretically, patients with a thin cornea preoperatively are felt to be more prone to iatrogenic keratectasia (4).

Because the posterior corneal surface is not affected by the corneal refractive procedure, assessing its stability is of great importance in identifying iatrogenic keratectasia. Posterior corneal elevation data, independent of orientation and axis, have been shown to be the most effective indicators for evaluating corneal stability, as well as for the diagnosis of iatrogenic keratectasia at an early stage (5–7).

Owing to the development of femtosecond laser technology, small incision lenticule extraction (SMILE), the flapless corneal refractive procedure using the femtosecond laser alone, leaves most of the anterior cornea tissue intact after surgery (8). Published investigations have shown that SMILE enables less corneal tensile strength loss than traditional corneal refractive surgeries (9). Additionally, we studied the biomechanical changes in thin corneas after SMILE and the results showed that SMILE could reduce the deterioration of corneal biomechanics (10). However, to the best of our knowledge, information regarding the long-term impact of SMILE on the posterior corneal surface of thin corneas is still lacking.

In the current study, we evaluated the posterior corneal stability of eyes with a thin cornea preoperatively, as well as moderate-thick corneas, for 3-years after SMILE, as well as compared changes in elevation among different groups.

## Patients and Methods

### Patients

In accordance with the tenets of the Declaration of Helsinki, the Ethics Committee of Fudan University Eye and the ENT Hospital Review Board (Shanghai, China) approved the study protocol. Written informed consent was obtained from each subject before their participation in the study.

In this prospective study, 97 eyes from 97 patients undergoing SMILE at the Department of Ophthalmology, Eye and ENT Hospital of Fudan University (Shanghai, People's Republic of China) were recruited. The patients had no ocular diseases other than refractive error and met the inclusion criteria. Patient inclusion criteria were as follows: age >18 years and stable refractive error in the preceding 2 years (SE changes < −1.00D), CDVA ≥20/25, sufficient corneal thickness (preoperative minimum central corneal thickness ≥480 μm and residual bed thickness >250 μm) and no use of soft contact lenses for >2 weeks, hard contact lenses for >1 month, and Ortho-K contact lenses for >3 months. Exclusion criteria were as follows: history of ocular surgery or trauma, systemic diseases, suspicion of keratoconus or dry eye, unrealistic expectations from refractive surgery. One random eye from each of the patients was selected.

All patients underwent a comprehensive preoperative ophthalmologic examination, including slit-lamp examination, and measurement of uncorrected distance visual acuity (UDVA), corrected distance visual acuity (CDVA), intraocular pressure, and Pentacam HR imaging (Oculus GmbH, Wetzlar, Germany).

### Surgical Procedure

The VisuMax femtosecond laser system (Carl Zeiss Meditec AG, Germany) was used to perform all surgeries. After applying topical anesthesia, the patient was positioned under the curved contact glass and was instructed to focus on the internal target light. The surgeon then achieved correct corneal centration and initiated the suction, followed by femtosecond laser scanning. Once the laser scanning was complete, the surgeon inserted a spatula into the cornea, dissected the lenticule interface, and manually extracted the lenticule. The femtosecond laser settings were as follows: repetition rate, 500 kHz; 120 μm intended cap thickness; 5.8 to 6.5 mm optical zone (lenticule diameter); 7.6 mm cap diameter; and a 2 mm side cut at the 12 o'clock point. The same experienced surgeon performed all the procedures (XZ).

### Pentacam Scheimpflug Imaging

All eyes were examined using the Pentacam HR imaging system. The patient was instructed to position their head on the headrest and focus on the target light. After attaining alignment, the device captured 25 images and automatically recorded 12,500 elevation points within 2 s. To avoid miscalculations of poor imaging, the quality of each measurement was shown in the specification window, and only results with “OK” statements were accepted. The examination was duplicated if the statement did not meet the requirements (marked yellow or red). Only maps with at least 10 mm of corneal coverage and no deduced data in the central 9-mm zone were accepted.

### Postoperative Examination

Follow-up appointments were scheduled 1 day and 3 years after the surgery. Postoperative examinations included Pentacam imaging examinations, slit-lamp examination, and measurement of UDVA and CDVA, spherical equivalent (SE) refraction, and intraocular pressure.

### Data Collection

Elevation data of the posterior corneal surfaces were acquired from the Pentacam images. The reference best-fit sphere (BFS) was defined in the central 8.0-mm region of the preoperative data so that it was the same across all images. For points above the reference, the values were positive; for points below the reference, the values were negative. The calculated values of single points were the posterior central elevation (PCE) and posterior elevation at the preoperative thinnest point (PTE) in the central 4-mm area above the BFS. The other 26 determined points in the central 6-mm zone were also obtained as follows: four points at a 1 mm distance from the center along the 45°, 135°, 225°, and 315° meridians (0° defined as the horizontal semi-meridian on the right, and rotating counterclockwise in both eyes), eight points at a 2 mm distance from the center at 0°, 45°, 90°, 135°, 180°, 225°, 270°, 315°, and 14 points at a distance of 3 mm from the center along 15°, 45°, 75°, 90°, 105°, 135°, 165°, 195°, 225°, 255°, 270°, 285°, 315°, and 345°. Posterior corneal elevation in the central 4 mm area (PCE-4 mm) and in various concentric circles (2 mm diameter, MPE-2 mm; 4 mm diameter, MPE-4 mm; 6 mm diameter, MPE-6 mm) was calculated as the mean value from points in the corresponding area. Twenty-four points in different optical zones (except two points from these 26 points: 2 mm distance from the center at 0° and 180°) were divided symmetrically into superior (MPE-S) and inferior (MPE-I) hemispheres by the 0° to 180° meridian. Changes in posterior elevation were determined by subtracting preoperative data from postoperative data (difference elevation map). The change in elevation was due to a shift of the posterior corneal surface. Elevation data were recorded in an Excel spreadsheet (Microsoft Corp, Redmond, WA, USA) for further analysis.

### Statistical Analysis

Descriptive results are presented as the mean and standard deviation. The Kolmogorov–Smirnov normality test and test for homogeneity of variances were performed for all data. The analysis of variance (ANOVA) for repeated measures with a Bonferroni correction was employed to compare pre- and post-operative values. If the data were not suitable for ANOVA analysis, we used the Friedman's rank test for k-correlated samples. The ANOVA test was performed to compare preoperative continuous data between groups; if the data were not suitable for ANOVA analysis, the Kruskal–Wallis test was used. Pearson's Chi-squared test was used to compare the preoperative classified data. A bivariate normal analysis was performed before the correlation test. Pearson's or Spearman's correlation test was used to determine the association between the changes in posterior corneal elevation and preoperative SE, preoperative minimum central corneal thickness (CCT), ablation depth (AD), and residual bed thickness (RBT). Statistical analyses were performed using SPSS version 20.0 (SPSS Inc., Chicago, IL, USA). Statistical significance was set at P < 0.05.

## Results

Eyes were categorized into the following groups based on CCT: group A (37 eyes; range, 480–499 μm, 491.14 ± 5.92 μm), group B (30 eyes; range, 500–529 μm, 508.73 ± 7.97 μm), group C (30 eyes; range, 530–560 μm, 544.00 ± 8.97 μm). All surgeries were completed successfully, and no complications occurred either during or after the procedure. Preoperative age, sex, sphere, cylinder, SE, and AD did not significantly differ among the three groups. Detailed information is presented in Table 1.

**Table 1:** Patient demographic information in different groups (mean ± standard deviation).

**Mean ±standard deviation (Range)**				
	**Group A** **(*****n*** **= 37)**	**Group B** **(*****n*** **= 30)**	**Group C** **(*****n*** **= 30)**	***P*** **-Value**
Age (y)	26.10 ± 5.97 (19 to 43)	27.47 ± 4.81 (19 to 36)	29.90 ± 7.82 (18 to 44)	0.101^b^
Gender (M/F)	15/22	7/23	7/23	0.199^a^
Preoperative sphere (D)	−5.07 ± 1.95 (−8.75 to −1.50)	−5.58 ± 1.20 (−8.00 to −3.50)	−5.34 ± 1.82 (−9.00 to −2.75)	0.439^b^
Preoperative cylinder (D)	−0.84 ± 0.92 (−4.50 to 0.00)	−0.67 ± 0.38 (−1.50 to 0.00)	−0.60 ± 0.41 (−1.50 to 0.00)	0.722^b^
Preoperative SE (D)	−5.49 ± 1.98 (−8.88 to −1.50)	−5.92 ± 1.18 (−8.25 to −4.00)	−5.64 ± 1.84 (−9.00 to −3.00)	0.578^b^
Preoperative CCT (μm)	491.14 ± 5.92 (480 to 499)	508.73 ± 7.97 (500 to 529)	544.00 ± 8.97 (530 to 560)	<0.001^b^
AD (μm)	109.03 ± 19.94 (63 to 136)	120.00 ± 16.40 (90 to 152)	111.53 ± 26.91 (71 to 164)	0.160^b^
RBT (μm)	282.11 ± 19.59 (252 to 325)	289.00 ± 17.03 (259 to 334)	332.47 ± 29.40 (280 to 384)	<0.001^b^
CDVA (LogMAR)	−0.07 ± 0.06 (0.00 to −0.20)	−0.07 ± 0.05 (0.00 to −0.20)	−0.11 ± 0.07 (0.00 to −0.20)	0.021^b^
Postoperative sphere (D)	−0.17 ± 0.44 (−1.50 to 0.75)	−0.13 ± 0.39 (−1.25 to 0.75)	−0.10 ± 0.46 (−1.50 to 0.50)	0.622^b^
Postoperative cylinder (D)	−0.21 ± 0.31 (−1.25 to 0.00)	−0.24 ± 0.27 (−1.00 to 0.00)	−0.20 ± 0.26 (−0.75 to 0.00)	0.515^b^
Postoperative SE (D)	−0.25 ± 0.44 (−1.50 to 0.375)	−0.26 ± 0.42 (−1.50 to 0.50)	−0.18 ± 0.43 (−1.50 to 0.50)	0.566^b^

*D, diopters; SE, spherical equivalent; CCT, minimum central corneal thickness; AD, ablation depth; RBT, residual bed thickness; CDVA, corrected distance visual acuity*.

a *The Pearson's chi-squared test*.

b *The Kruskal-Wallis test*.

### Visual Outcomes

**Figure 1:**
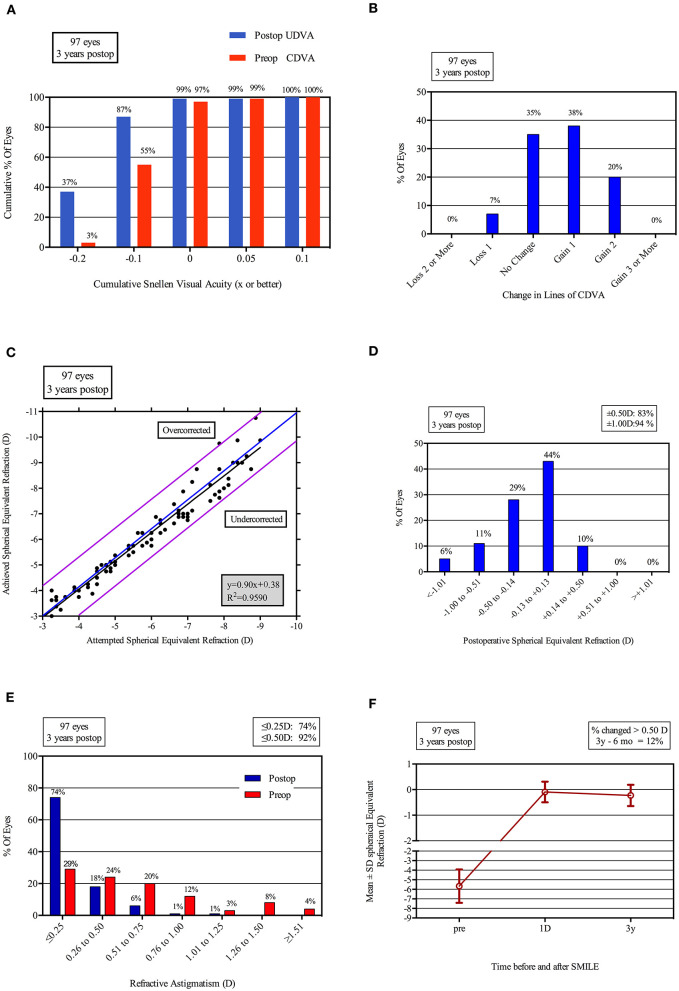
Refractive outcomes at 3 years postoperatively in 97 eyes after SMILE. UDVA, uncorrected distance visual acuity (LogMAR); CDVA, corrected distance visual acuity (LogMAR); D, diopters; Postop, postoperative; Preop, preoperative. **(A)** Uncorrected distance visual acuity; **(B)** Change in corrected distance visual acuity; **(C)** Spherical equivalent attempted vs achieved; **(D)** Spherical equivalent refractive accuracy; **(E)** Refractive astigmatism; **(F)** Stability of spherical equivalent refraction.

The safety index and efficacy index were 1.14 and 1.03, respectively, in group A, 1.16 and 1.11 in group B, and 1.18 and 1.03 in group C. At the 3-year follow-up, 97% (36/37) of all eyes in group A, 100% (30/30) of eyes in group B, and 97% (29/30) in group C had an UDVA of 0.1 or better (LogMar). No eyes lost two or more lines of CDVA in any of the groups. No statistical difference was found among three groups. In each group, 54% (20/37 in group A), 63% (19/30 in group B), and 57% (17/30 in group C) of the treated eyes gained one or more lines of CDVA. All of the eyes were within ± 1.50 D and 95% of the eyes were within ± 1.00 D. In group A, 81% (30/37) of the eyes were within ± 0.5 D. For group B and group C, 83% (25/30) and 87% (26/30) were within ± 0.5 D, respectively (Figure 1).

### Posterior Corneal Elevation Changes

#### Group A

**Figure 2:**
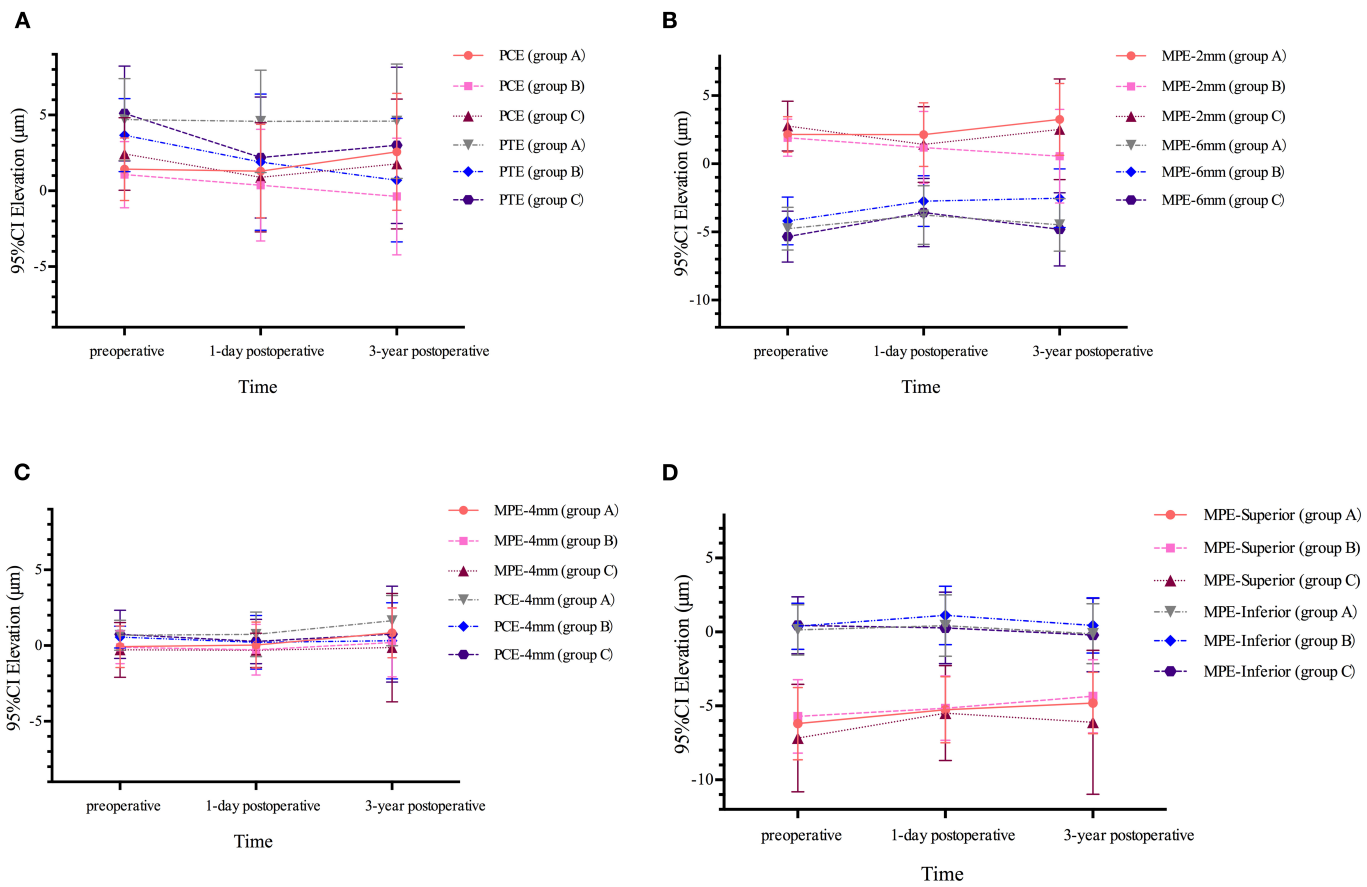
Posterior corneal elevation before and after SMILE in three groups. **(A)** Posterior elevation in central (PCE) and preoperative thinnest point (PTE) in three groups. **(B)** Posterior elevation in 2 mm diameter (MPE-2 mm) and 6 mm diameter (MPE-6 mm) in three groups. **(C)** Posterior elevation in 4 mm diameter (MPE-4 mm) and central 4 mm area (PCE-4 mm) in three groups. **(D)** Posterior elevation in superior hemisphere (MPE-Superior) and inferior hemisphere (MPE-Inferior) in three groups.

As shown in Table 2 and Figure 2, for single variables, PCE and PTE showed no statistically significant changes after the 3-year observation period following SMILE. (*P* > 0.125) A slight forward change was exhibited in all three concentric circles; however, a statistical difference was only noted for MPE-4 mm between baseline and after the 3 years (*P* = 0.005). Although the degree of posterior elevation of three areas (PCE-4 mm, MPE-Superior, and MPE-Inferior) displayed similar tiny forward displacements during the follow-up period, MPE-Inferior did not significantly differ between baseline and after 3 years (*P* > 0.436); the PCE-4 mm and MPE-Superior values after 3 years were greater than the preoperative values (*P* ≤ 0.013).

**Table 2:** Posterior corneal elevation before and after SMILE in different groups.

		**Time point**			***P*** **-value**			
		**Preop**	**Postop 1 d**	**Postop 3 y**		**Preop-1 d**	**Preop-3y**	**Postop1d-3y**
Group A	PCE	1.43 ± 2.06	1.30 ± 3.08	2.57 ± 3.86	0.125^b^			
	PTE	4.70 ± 2.70	4.58 ± 3.37	4.59 ± 3.78	0.577^b^			
	MPE-2 mm	2.16 ± 1.30	2.14 ± 2.34	3.26 ± 2.64	0.283^b^			
	MPE-4 mm	−0.07 ± 1.37	0.04 ± 1.51	0.85 ± 1.65	0.002^a^	1.000	0.005	0.023
	MPE-6 mm	−4.76 ± 1.57	−3.76 ± 2.15	−4.48 ± 1.93	0.000^a^	0.000	0.405	0.038
	PCE-4 mm	0.68 ± 0.99	0.74 ± 1.47	1.65 ± 1.66	0.003^a^	1.000	0.013	0.035
	MPE-Superior	−6.20 ± 2.44	−5.26 ± 2.24	−4.80 ± 2.07	0.000^b^	0.001	0.000	0.419
	MPE-Inferior	0.14 ± 1.70	0.43 ± 2.08	−0.12 ± 2.03	0.436^b^			
Group B	PCE	1.07 ± 2.18	0.37 ± 3.68	−0.37 ± 3.85	0.517^b^			
	PTE	3.67 ± 2.41	1.89 ± 4.49	0.70 ± 4.07	0.004^b^	0.062	0.008	1.000
	MPE-2 mm	1.91 ± 1.36	1.20 ± 2.64	0.56 ± 3.43	0.266^b^			
	MPE-4 mm	−0.12 ± 1.08	−0.27 ± 1.67	0.20 ± 2.26	0.234^b^			
	MPE-6 mm	−4.19 ± 1.75	−2.74 ± 1.86	−2.53 ± 2.15	0.000^a^	0.000	0.000	1.000
	PCE-4 mm	0.56 ± 0.72	0.22 ± 1.77	0.32 ± 2.51	0.936^b^			
	MPE-Superior	−5.71 ± 2.48	−5.15 ± 2.17	−4.35 ± 2.47	0.018^a^	0.388	0.021	0.506
	MPE-Inferior	0.39 ± 1.56	1.12 ± 1.97	0.44 ± 1.87	0.125^a^			
Group C	PCE	2.43 ± 2.39	0.89 ± 3.60	1.77 ± 4.29	0.066^a^			
	PTE	5.10 ± 3.13	2.20 ± 3.99	3.00 ± 5.15	0.000^a^	0.000	0.024	0.788
	MPE-2 mm	2.77 ± 1.82	1.42 ± 2.77	2.53 ± 3.69	0.008^b^	0.007	0.467	0.320
	MPE-4 mm	−0.28 ± 1.81	−0.31 ± 1.13	−0.13 ± 3.58	0.332^b^			
	MPE-6 mm	−5.34 ± 1.87	−3.58 ± 2.50	−4.81 ± 2.69	0.000^b^	0.000	0.029	0.467
	PCE-4 mm	0.74 ± 1.59	0.27 ± 1.47	0.76 ± 3.16	0.331^b^			
	MPE-Superior	−7.18 ± 3.63	−5.49 ± 3.21	−6.11 ± 4.87	0.000^b^	0.000	0.000	1.000
	MPE-Inferior	0.45 ± 1.93	0.28 ± 2.42	−0.21 ± 2.48	0.051^a^			

*SMILE, small incision lenticule extraction; PCE, posterior central elevation; PTE, posterior elevation at the preoperative thinnest point in the central 4-mm area; MPE-2 mm, mean posterior corneal elevation in the 2-mm optical zone as a function of the meridian of four points; MPE-4 mm, mean posterior corneal elevation in the 4-mm optical zone as a function of the meridian of eight points; MPE-6 mm, mean posterior corneal elevation in the 6-mm optical zone as a function of the meridian of 14 points; PCE-4 mm, mean posterior corneal elevation in the central 4-mm zone of 13 points; MPE-Superior, mean posterior corneal elevation in the superior area; MPE-Inferior, mean posterior corneal elevation in the inferior area*.

a *The analysis of variance (ANOVA) with the Bonferroni correction*.

a * Kruskal-Wallis test*.

#### Group B

The PCE and PTE data both displayed a backward trend after the procedure, and a significant statistical difference was found for the PTE (PTE, *P* = 0.004; PCE, *P* = 0.517). From the 2 mm circle to the 6 mm circle, the backward displacement was gradually converted to the forward change; a significant difference was observed in the 6-mm diameter (*P* < 0.001). The MPE-superior value was significantly increased after 3 years of SMILE compared to the preoperative value (*P* = 0.021). No statistically significant differences were observed in the PCE-4 mm or MPE-inferior data between different follow-up visits (*P* ≥ 0.125) (Table 2, Figure 2).

#### Group C

The change in posterior corneal elevation in thick corneas was similar to that in the moderate-thick cornea group (Table 2, Figure 2). Both PCE and PTE after SMILE were lower than the preoperative values, and a statistical difference was found for the PTE (PTE, *P* < 0.001; PCE, *P* = 0.006). The value of the 2-mm optical zone decreased while the 4-mm and 6-mm optical zones were elevated after 3 years (MPE-2 mm, *P* = 0.008; MPE-4 mm, *P* = 0.332; MPE-6 mm, *P* < 0.001). There was a significant difference in MPE-6 mm and MPE-Superior between baseline and after 3 years (*P* < 0.001).

All changes in posterior corneal elevation 3 years after SMILE in the three groups were minimal, and the results are summarized in Table 3. The mean changes in PCE and MPE-2 mm in group A were small positive values, whereas they were negative in group B and group C. The MPE-6 mm results were contrary to those of PCE and MPE-2 mm. The differences in PCE, MPE-2 mm, and MPE-6 mm were only statistically significant between group A and group B (For PCE and MPE-2 mm, changes in group A were more than that in group C; changes of MPE-6 mm in group C were greater than group A. *P* ≤ 0.040). PTE in the three groups exhibited consistently backward displacement, and a statistically significant difference was observed between group A and group B (*P* = 0.028). There was no significant difference in the other calculated values among the three groups.

**Table 3:** Changes of posterior corneal elevation after 3 years of SMILE in different groups.

	**Group A**	**Group B**	**Group C**	***P*** **-value**			
					***P12***	***P13***	***P23***
PCE	1.14 ± 3.40	−1.43 ± 3.81	−0.67 ± 3.01	0.017^b^	0.040	0.052	1.000
PTE	−0.11 ± 3.20	−2.97 ± 3.76	−2.10 ± 4.03	0.027^b^	0.028	0.211	1.000
MPE-2 mm	1.10 ± 2.46	−1.35 ± 3.31	−0.24 ± 2.89	0.003^a^	0.002	0.181	0.416
MPE-4 mm	0.92 ± 1.43	0.32 ± 1.81	0.15 ± 3.61	0.486^b^			
MPE-6 mm	−0.27 ± 1.24	1.66 ± 2.01	0.53 ± 1.78	0.017^b^	0.014	1.000	0.175
PCE-4 mm	0.98 ± 1.67	−0.24 ± 2.24	0.02 ± 3.09	0.191^b^			
MPE-Superior	1.40 ± 1.78	1.36 ± 2.29	1.08 ± 3.45	0.909^b^			
MPE-Inferior	−0.26 ± 1.23	0.06 ± 1.63	−0.66 ± 1.44	0.232^b^			

*SMILE, small incision lenticule extraction; PCE, posterior central elevation; PTE, posterior elevation at the preoperative thinnest point in the central 4-mm area; MPE-2 mm, mean posterior corneal elevation in the 2-mm optical zone as a function of the meridian of 4 points; MPE-4 mm, mean posterior corneal elevation in the 4-mm optical zone as a function of the meridian of eight points; MPE-6 mm, mean posterior corneal elevation in the 6-mm optical zone as a function of the meridian of 14 points; PCE-4 mm, mean posterior corneal elevation in the central 4-mm zone of 13 points; MPE-Superior, mean posterior corneal elevation in the superior area; MPE-Inferior, mean posterior corneal elevation in the inferior area; P12, comparison between thin and moderate-thick cornea group; P13, comparison between thin and thick cornea group; P23, comparison between moderate-thick and thick cornea group*.

a *The analysis of variance (ANOVA) with the Bonferroni correction*.

b *The Kruskal-Wallis test*.

### Correlations

In group A, changes in PCE, PTE, MPE-2 mm, MPE-6 mm, PCE-4 mm, MPE-Superior, and MPE-Inferior demonstrated significant statistical correlations with preoperative SE, preoperative CCT, AD, and RBT (all *P* ≤ 0.032). The correlation was negative for SE, CCT, and RBT, and was positive for AD (except for the inferior area, the 4-mm and 6-mm optical zone, *P* ≤ 0.032). Changes in PCE and PTE in group B were only found to be significantly negatively correlated with preoperative SE (*P* < 0.001). Significant relationships were also noted between changes in MPE-6 mm, MPE-Superior, MPE-Inferior, and RBT in group B (*P* < 0.001). The relationship between changes in the calculated values and preoperative SE, CCT, AD, and RBT was also significant in group C (except for PCE, *P* ≤ 0.045). The statistical results of the correlation tests for the various groups are displayed in Table 4.

**Table 4:** Statistical correlation between changes of posterior corneal elevation after 3 years of SMILE and preoperative spherical equivalent (SE), preoperative minimum central corneal thickness (CCT), ablation depth (AD), residual bed thickness (RBT).

		**SE**		**CCT**		**AD**		**RBT**	
		***r***	***P***	***r***	***P***	***r***	***P***	***r***	***P***
Group A	PCE	−0.222	0.029	−0.268	0.008	0.218	0.032	−0.278	0.006
	PTE	−0.829	0.000	−0.651	0.000	0.645	0.000	−0.768	0.000
	MPE-2 mm	−0.664	0.000	−0.789	0.000	0.646	0.000	−0.715	0.000
	MPE-4 mm	−0.087	0.395	−0.472	0.000	0.123	0.231	−0.140	0.170
	MPE-6 mm	0.513	0.000	0.510	0.000	−0.427	0.000	0.468	0.000
	PCE-4 mm	−0.301	0.003	−0.599	0.000	0.323	0.001	−0.361	0.000
	MPE-Superior	−0.587	0.000	−0.359	0.000	0.624	0.000	−0.628	0.000
	MPE-Inferior	0.714	0.000	0.428	0.000	−0.661	0.000	0.676	0.000
Group B	PCE	−0.781	0.000	−0.019	0.857	0.183	0.072	−0.183	0.072
	PTE	−0.747	0.000	−0.027	0.793	0.126	0.219	−0.126	0.219
	MPE-2 mm	−0.147	0.150	0.092	0.369	0.088	0.392	−0.094	0.357
	MPE-4 mm	−0.634	0.000	0.320	0.001	−0.180	0.078	0.180	0.078
	MPE-6 mm	0.014	0.894	−0.255	0.012	0.451	0.000	−0.451	0.000
	PCE-4 mm	−0.621	0.000	0.183	0.073	−0.134	0.191	0.134	0.191
	MPE-Superior	−0.637	0.000	0.118	0.249	0.373	0.000	−0.373	0.000
	MPE-Inferior	−0.603	0.000	−0.641	0.000	0.513	0.000	−0.513	0.000
Group C	PCE	−0.157	0.125	−0.362	0.000	0.118	0.252	−0.307	0.002
	PTE	0.453	0.000	−0.421	0.000	−0.579	0.000	0.368	0.000
	MPE-2 mm	0.272	0.007	−0.204	0.045	−0.384	0.000	0.281	0.005
Group C	MPE-4 mm	0.478	0.000	−0.269	0.008	−0.566	0.000	0.523	0.000
	MPE-6 mm	0.645	0.000	−0.369	0.000	−0.535	0.000	0.351	0.000
	PCE-4 mm	0.470	0.000	−0.266	0.008	−0.563	0.000	0.512	0.000
	MPE-Superior	0.652	0.000	−0.701	0.000	−0.659	0.000	0.310	0.002
	MPE-Inferior	−0.885	0.000	0.312	0.002	0.818	0.000	−0.659	0.000

*SMILE, small incision lenticule extraction; PCE, posterior central elevation; PTE, posterior elevation at the preoperative thinnest point in the central 4-mm area; MPE-2 mm, mean posterior corneal elevation in the 2-mm optical zone as a function of the meridian of four points; MPE-4 mm, mean posterior corneal elevation in the 4-mm optical zone as a function of the meridian of eight points; MPE-6 mm, mean posterior corneal elevation in the 6-mm optical zone as a function of the meridian of 14 points; PCE-4 mm, mean posterior corneal elevation in the central 4-mm zone of 13 points; MPE-Superior, mean posterior corneal elevation in the superior area; MPE-Inferior, mean posterior corneal elevation in the inferior area*.

## Discussion

The conventional view is that a thin cornea, defined as a minimum CCT thinner than 500 μm, is one of the risk factors for developing keratectasia after corneal refractive surgery (11–13). However, it is still unclear whether eyes with a thin cornea are more susceptible to this severe post-complication after SMILE as there is insufficient data. Thus, in the current study, we reported the refractive results and corneal stability of thin corneas after SMILE and compared them with moderate-thick corneas.

Previously, de Benito-Llopis evaluated the 10-year outcomes of excimer laser surface ablation for thin corneas and reported a safety index of 0.9 and an efficacy index of ~0.8 (13, 14). For thin corneas that underwent LASIK, the 5-year safety and efficacy indexes were 1.09 and 0.99, respectively, as reported by Song (12). The percentages of eyes that achieved a postoperative SE within ± 1.0 D and ± 0.50 D were <90% and 80%, respectively (12, 13, 15). In the current study, all three groups showed high levels of safety and effectiveness: the safety index was higher than 1.1 and the efficacy index was better than 1.0. Furthermore, 95% of all eyes were within ± 1.0 D and 81% (30/37) of eyes in the thin cornea group were within ± 0.5 D. Compared with the aforementioned studies, we found that SMILE could offer similar and even better refractive outcomes than traditional surgeries for thin cornea correction.

As shown in the Tables 2 and 3, two determined single variables were investigated, and the results showed some differences: (1) PCE and PTE showed no statistically forward displacement in any of the three groups. (2) The change of PCE after 3 years was small positive value in group A, while were negatives values in the other two groups presenting a slight backward trend. (3) PTE remained stable in group A, but showed a descending tendency and exited statistical difference in group B and C. This is not the first time that such decreasing changes in these two variables have been discovered. In our previous investigation of eyes that underwent SMILE with a corneal thickness of more than 500 μm, PCE and PTE also showed decreasing changes of −2.39 ± 2.85 μm and −2.33 ± 2.90 μm after 3 years, respectively (7). Zhou et al. studied the variations of PCE and PTE 2 years after SMILE in a high myopia group, and the results also displayed backward displacements (16). Comparable outcomes were noted after LASIK and the surface ablation technique as well (17, 18). All these abovementioned studies evaluating the posterior surface in eyes with corneal thickness more than 500 μm and the results were consistent with ours in group B and C. However, it is important to note that in patients with corneal thickness thinner than 500 μm, PCE showed tiny forward change (1.14 ± 3.40 μm) though the result was not statistically significant. Whether the results were clinically meaningful is not clear, suggesting further observations are required to reveal more consequences.

PCE-4 mm, MPE-Superior, and MPE-Inferior were the calculated values that we studied to represent the changes in the posterior corneal surface in a certain area. Among these three values, PCE-4 mm and MPE-Inferior were more important for most keratectasia cases with corneal protrusion in the central or inferior areas as initial signs. In group A, we found a significant change in PCE-4 mm 3 years after SMILE. However, the data were only 0.98 ± 1.67 μm, which is very small; it was not higher than that in the other two groups. Additionally, MPE-inferior yielded no increasing changes in any of the three groups. A handful of studies assessing PCE-4 mm change after SMILE in corneas thicker than 500 μm showed some subtle distinctions: Wu and other researchers observed a decreasing change of ~1 μm, whereas the change was more pronounced in another study (0.29 ± 2.77 μm) (19–21). All these elevation changes were very small and investigators commented that iatrogenic corneal protrusion could not be deduced from these outcomes (22, 23). Based on these published articles and our results, it is reasonable to conclude that no forward corneal shift occurs in thin corneas in the 3 years following SMILE.

Interestingly, space-dependent alterations in posterior corneal elevation were observed when analyzing various concentric annuluses. In the 2- and 4-mm zones, the posterior corneal elevation remained stable or showed a minor decrease; conversely, the elevation data were elevated in the peripheral area (6 mm zone), thus indicating subtle forward displacement in this area. Previously, Zhang conducted a detailed study of the data on posterior corneal elevation changes following two types of representative traditional excimer laser surgery: LASIK and epithelia LASIK (epi-LASIK) (18). According to their results, both LASIK and epi-LASIK induced a backward shift or flattening in the central area, and slight forward displacement in the peripheral area. Another published article compared posterior corneal elevation after SMILE for different cap thicknesses (21). Although the study area was limited to the 2-mm and 4-mm diameter annulus and lacked data in the peripheral area, the results were in agreement with ours. The hyperopic shift model proposed by Dupps and Roberts may provide some explanations for this phenomenon (24). As the designed optical zone is typically around 6 mm, the corneal lamella in this area is removed during the procedure, thus leading to peripheral lamellae relaxation. Subsequently, swelling and redistribution of corneal tension result in central corneal flattening and peripheral steepening. It is noteworthy that some confounding factors, namely postoperative responses and a lower intraocular pressure than that preoperatively, may also play a role in causing the alteration (18, 25).

The correlation tests between AD, RBT, and changes in posterior corneal elevation showed vastly different outcomes in various groups. In group A, most elevation changes were found to be positively correlated with AD and negatively correlated with RBT. However, in group C, the opposite results were observed. These statistically significant relationships rarely existed in group B. The results of our study are inconsistent with those of previous studies. Khairat evaluated posterior corneal elevation changes after LASIK and reported no correlation between MPE-4 mm and RBT (26). A linear relationship between changes in PCE, MPE-2 mm, MPE-4 mm, PCE-4 mm, and RBT after SMILE has not been documented in other published articles (21). The following reasons may account for this difference, among which the foremost concern is the precise categorization in the current study. We divided all the eyes into three groups based on preoperative corneal thickness, which other studies have seldom done before. Owing to the strict classification, the impact of corneal thickness on correlation tests could be eliminated to the best extent; moreover, the accuracy and efficacy of the tests were also improved in this way. Another minor factor may be the follow-up time: the two abovementioned articles had short follow-up periods (3 months); other studies yielded no linear relationships for no more than 18 months (18, 22, 27). In light of our results, the higher risk of postoperative ectasia in thin corneas with lower RBT should not be ignored.

From a statistical point of view, ectasia occurs mostly at an average of 13 to 15 months after corneal refractive surgery (28). Hence, the patients in the current study were followed for more than 15 months, and no clinical ectasia developed during the 3-year observation period. In addition, no forward displacement of the posterior corneal elevation was observed in the correction of thin corneas. Several researchers have observed the long-term outcomes of eyes with thin corneas after excimer laser surgery and concluded that corneal refractive surgery seems to be safe and effective with a strict preoperative assessment (13, 15, 29, 30). Randleman summarized all the reported cases of iatrogenic ectasia in 7 years and stated that preoperative abnormal topography was the main cause of ectasia, followed by less RBT (28). On the basis of published investigations, researchers suggested that an RBT should not be thinner than 250 μm to ensure surgical safety, especially in patients with thin corneas (13, 31). In our study, we strictly excluded patients with any abnormality using Pentacam Scheimpflug imaging (32), and no eyes had an RBT thinner than 250 μm. It is worth mentioning that Reinstein estimated postoperative stromal tensile strength after corneal laser surgery (photorefractive keratectomy (PRK), LASIK, and SMILE) and found that the stromal strength was strongest after SMILE because the anterior lamellae were intact (9). Despite this promising result, we strongly recommend caution when executing SMILE with thin corneas.

Limitations of the current study were that only 97 eyes were included in this study, observation data from a larger sample are required to verify the results in the future. Additionally, optical zone was varied due to the patient's scotopic pupil in order to optimize the postoperative visual quality, which may have indirect impact on the results. Along with the findings of this study, further investigations of different patient databases may provide more information on corneal surface stability after SMILE in thin corneas.

In conclusion, with a strict preoperative assessment, SMILE was associated with high levels of safety and efficacy in correcting myopia in thin corneas and enabled a stable posterior corneal surface over a 3-year follow-up period.

## Data Availability Statement

The original contributions presented in the study are included in the article/supplementary material, further inquiries can be directed to the corresponding author.

## Ethics Statement

The Ethics Committee of Fudan University Eye and ENT Hospital Review Board (Shanghai, China) approved the study protocol. The patients/participants provided their written informed consent to participate in this study.

## Author Contributions

YZ, DF, and XZ: study concept and design. YZ, DF, and ZC: data collection. YZ: data analysis, interpretation and drafting of the manuscript. YZ, DF, ZC, and XZ: critical revision of the manuscript. XZ: supervision. All authors read and approved the final manuscript.

## Funding

This work was supported by Shanghai Sailing Program (Grant No. 20YF1405200), The National Natural Science Foundation of China (Grant No. 82000932), The National Natural Science Foundation of China (Grant No. 81770955), Project of Shanghai Science and Technology (Grant No. 20410710100), Joint Research Project of New Frontier Technology in Municipal Hospitals (Grant No. SHDC12018103), Clinical Research Plan of SHDC (Grant No. SHDC2020CR1043B), and Project of Shanghai Xuhui District Science and Technology (2020-015).

## Conflict of Interest

The authors declare that the research was conducted in the absence of any commercial or financial relationships that could be construed as a potential conflict of interest.

## Publisher's Note

All claims expressed in this article are solely those of the authors and do not necessarily represent those of their affiliated organizations, or those of the publisher, the editors and the reviewers. Any product that may be evaluated in this article, or claim that may be made by its manufacturer, is not guaranteed or endorsed by the publisher.
